# ATPase Valosin-Containing Protein (VCP) Is Involved During the Replication and Egress of Sialodacryoadenitis Virus (SDAV) in Neurons

**DOI:** 10.3390/ijms252111633

**Published:** 2024-10-29

**Authors:** Michalina Bartak, Weronika D. Krahel, Marcin Chodkowski, Hubert Grel, Jarosław Walczak, Adithya Pallepati, Michał Komorowski, Joanna Cymerys

**Affiliations:** 1 Division of Microbiology, Department of Preclinical Sciences, Institute of Veterinary Medicine, Warsaw University of Life Sciences, Ciszewskiego 8 St., 02-786 Warsaw, Poland; weronika_krahel@sggw.edu.pl; 2 Division of Medical and Environmental Microbiology, Military Institute of Hygiene and Epidemiology, Kozielska 4 St., 01-063 Warsaw, Poland; marcin.chodkowski@wihe.pl; 3 Department of Physics and Biophysics, Institute of Biology, Warsaw University of Life Sciences, 02-787 Warsaw, Poland; hubert_grel@sggw.edu.pl; 4 Department of Biosystems and Soft Matter, Institute of Fundamental Technological Research, Polish Academy of Sciences, Pawińskiego 5B St., 02-106 Warsaw, Poland; jwalczak@ippt.pan.pl (J.W.); apallepati@iimcb.gov.pl (A.P.); mkomor@ippt.pan.pl (M.K.); 5 Laboratory of Single-Molecule Biophysics, International Institute of Molecular and Cell Biology in Warsaw, Ks. Trojdena 4 St., 02-109 Warsaw, Poland

**Keywords:** SDAV, VCP, primary neurons, virion assembly, ERAD, eeyarestatin I

## Abstract

Sialodacryoadenitis virus (SDAV) has been identified as the etiological agent responsible for the respiratory system and salivary gland infections in rats. The existing literature on SDAV infections is insufficient to address the topic adequately, particularly in relation to the central nervous system. In order to ascertain how SDAV gains access to neuronal cells and subsequently exits, our attention was focused on the small molecule valosin-containing protein (VCP), which is an ATPase. VCP is acknowledged for its function in the ubiquitin-mediated proteasomal degradation of proteins, including those of viral origin. To ascertain the potential influence of VCP on SDAV replication and egress, high-content screening was employed to determine the viral titer and protein content. Western blot analysis was employed to ascertain the relative expression of VCP. Real-time imaging of SDAV-infected cells and confocal imaging for qualitative morphological analysis were conducted. The Eeyarestatin I (EerI) inhibitor was employed to disrupt VCP involvement in the endoplasmic reticulum-associated protein degradation pathway (ERAD) in both pre- and post-incubation systems, with concentrations of 5 μM/mL and 25 μM/mL, respectively. We demonstrated for the first time that SDAV productively replicates in cultured primary neurons. VCP expression is markedly elevated during SDAV infection. The application of 5 μM/mL EerI in the post-treatment system yielded a statistically significant inhibition of the SDAV yield. It is likely that this modulates the efficacy of virion assembly by arresting viral proteins in the submembrane area.

## 1. Introduction

Rodents are the most diverse and most abundant order of mammals in the world; they account for about 43% of mammalian species. Belonging to rodents, rats mainly inhabit urban areas, which is associated with their frequent interaction with humans. These animals are reservoirs of viruses, including coronaviruses, and thus pose a potential threat of zoonotic transmission. To date, two human-infecting coronaviruses, HCoV-HKU1 and HCoV-OC43, have been reported that are likely derived from rodent coronaviruses [1,2]. Following the One Health approach, viruses that cause rat infections should be studied more to better understand the nature of coronaviruses that are potentially dangerous to humans [3,4]. Sialodacryoadenitis virus (SDAV) is the aetiological agent of frequent respiratory infections in laboratory rats [5,6,7]. Clinical signs of infection include salivary and lacrimal gland inflammation and pharyngitis, photophobia, intermandibular edema, and, in some cases, reduced fertility. SDAV is antigenically related to the mouse hepatitis virus (MHV) serogroup [8] and can cause CNS infection and encephalopathies (documented only by in vivo studies) [9,10].

The viral replication cycle is a complex process in which the interactions of pathogen structures with host cell components are crucial. Identifying the proteins and other molecules present in cells that are important during viral entry, multiplication, and release are critical regarding potential zoonotic transfer. One protein that has been extensively studied in the context of viral infections due to its ubiquitous presence in cells and involvement in many cellular processes is valosin-containing protein (VCP). VCP belongs to the family of ATPases associated with diverse cellular activities (AAA+). It is expressed in different cells and is a highly conserved protein in all eukaryotes. It also occurs under other names—p97 in mammals, Cdc48 in yeast, CDC-48 in *Caenorhabditis elegans*, and Ter94 in *Drosophila melanogaster* [11,12,13]. It is mainly localized in the cytosol but is also present on the membranes of organelles such as endosomes, cell nucleus, endoplasmic reticulum, and Golgi apparatus [14]. VCP participates in numerous cellular processes, and the full range of VCP-related functions is still emerging [11]. VCP’s segregase activity and role in targeting proteins for degradation are best characterized. Both processes make essential contributions to maintaining cellular homeostasis. Eliminating abnormal proteins, organelles, and granular compartments is crucial for the proper function of neurons [15,16,17,18]. VCP also participates in endoplasmic reticulum-associated degradation (ERAD) [19], ribosome-associated protein quality control (RQC) [20], and mitochondria-associated degradation (MAD) [21]. The VCP is a promising target for clinical research in cancer treatment [22,23]. Although further research is necessary, recent reports indicate that the VCP inhibitor Eeyarestatin I (EerI) has shown promising results in the treatment of multiple myeloma and acute myeloid leukemia. In particular, the EerI-derived compound CB-5083 has demonstrated favourable synergistic effects when combined with other anti-leukemia drugs, including cytarabine and venetoclax [24]. This targeted therapy may be primarily based on the induction of apoptosis in tumour cells and the unfolded protein replication (UPR) pathway [22,25]. Another compound, VP20, has been demonstrated to inhibit tumour progression by inhibiting the NF-κB signalling pathway in the context of malignant multiple myeloma [26]. Another finding proved that targeting VCP with EerI in non-small cell lung carcinoma (NSCLC) restored p53 and NFB levels and ameliorated the growth and tumorigenicity, improving clinical outcomes [27]. In the case of experimental treatment of idiopathic epilepsy, VCP inhibition by EerI without stress induction, together with folding enhancement, represents a new strategy to restore the proteostasis of misfolding-prone GABAA receptors [28].

Considering the numerous functions performed in the cell and the widespread occurrence of VCP, the involvement of this protein during viral infections has begun to be studied. VCP has been shown to be utilized by various families of viruses, both with genomes in the form of RNA and DNA, during entry, replication, and exit from cells [29,30,31,32]. The protein likely mediates endosomal vesicle fusion through interactions with early endosome antigen 1 (EEA1), clathrin, and syntaxin 5 [33,34,35]. It is also possible that VCP controls the multimerization state of viral proteins during entry into the host cell [29,36]. The way viruses likely utilize VCP during the initial stages of infection has been best described in the family *Flaviridae*, whose cell entry is mediated by clathrin- and dynamin-dependent endocytosis [37,38,39,40]. VCP was also shown to be involved in the release of gammacoronavirus Infectious Bronchitis virus (IBV) and Human alphacoronavirus 229E (HCoV-229E) from endosomes [41]. The application of VCP-specific siRNA resulted in the accumulation of these viruses in the early endosomes of infected cells. This suggests the involvement of VCP in the acidification of the environment for envelope–membrane fusion and in the reprocessing and degradation of the nucleocapsid protein, which may indicate an important role for VCP in the infection of all coronaviruses [29,42]. Another process in which VCP is involved is the viral replication in cells. A disrupted replication process due to VCP inhibition has been observed in the case of infection with viruses of the *Togaviridae* family, including Chikunguya virus (CHIKV), Semliki Forest virus (SFV), and o’nyong’nyong virus (ONNV), or in case of flavivirus, Zika virus [40,43]. Studies indicate that VCP inhibition at different time points during infection with the human coronaviruses HCoV-229E and HCoV-OC43 resulted in reduced levels of RNA replication. In addition, these coronaviruses were found to affect proteins involved in the cell cycle through VCP [44]. Reduced replication levels were also observed when SARS-CoV-2-infected cells were treated with a VCP inhibitor [45]. The impact of VCP function in the late stage of infection during viral release was studied using the Rift Valley Fever virus (RVFV). RVFV has been found to utilize VCP, probably when moving viral glycoproteins into the Golgi apparatus and releasing virions from cells [46].

As mentioned above, VCP plays a role in the viral infection cycle at various stages, including receptor binding and entry, replication, and viral egress. This is primarily through its enzymatic activity. In addition, viruses can use VCP to exploit innate and adaptive immune responses, leading to chronic infections and virus-induced diseases [29]. The ubiquitous presence of VCP in cells, coupled with its involvement in the replication cycle of viruses from several families, including *Coronaviridae*, suggests that VCP may also be a critical factor during SDAV infection. It is essential to understand the molecular mechanisms underlying the potential proviral role of VCP, including the proteins that interact with SDAV, in order to develop effective therapies that target functions that inhibit the proviral action of VCP. The precise role of VCP during SDAV replication in cells, particularly in neurons, remains to be fully elucidated. In the research presented here, we have taken on this task.

## 2. Results

### 2.1. SDAV Cytopathic Effect in Cultured Neurons

**Figure 1:**
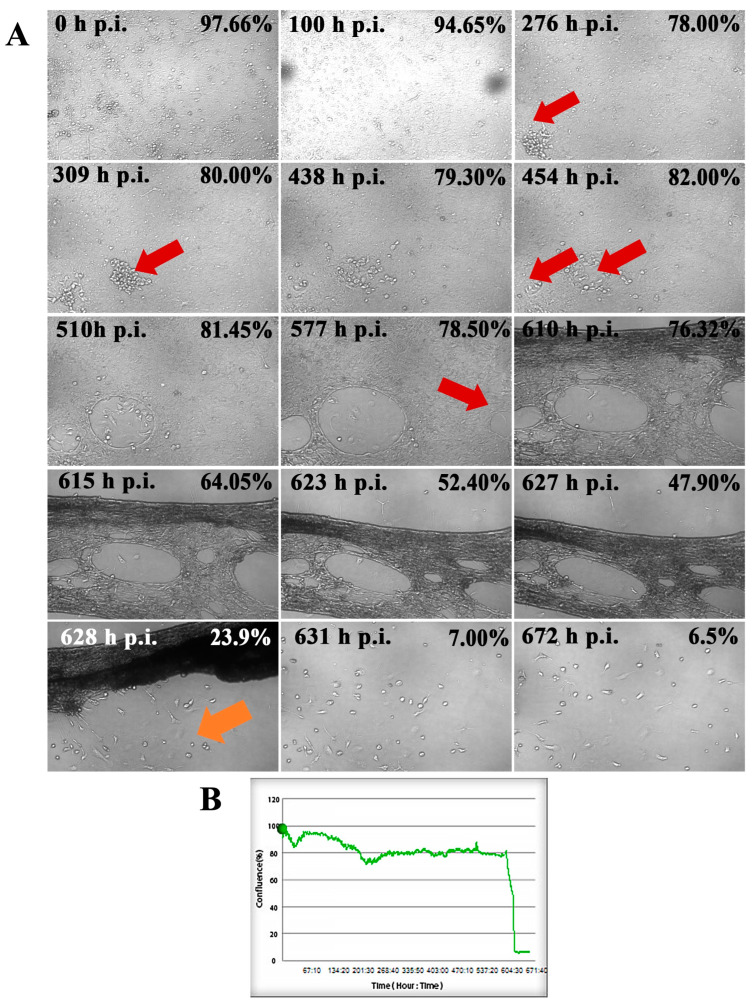
Real-time cell growth analysis of SDAV-infected primary murine neurons performed by live image movie analyzer JuLi™Br (NanoEnTek, Seoul, Republic of Korea). Cultures were observed for 672 h post-infection (h p.i.). Red arrows indicate the process of creating the cytopathic effect (CPE) in the form of plaques. The Orange arrow represents surviving neurons (**A**). The generated graph shows the percentage of cells’ confluence level [%] during the whole analysis [hours]. Images were taken every 10 min and analyzed for monolayer confluence (**B**). Objective magnification ×40.

An analysis of primary neuronal cultures of BALB/c mice showed that SDAV infection causes focal and diffuse cytopathic effects in the neurons, during which the cell shape changes and vacuolization and lysis occur, resulting in the formation of plaques. This gradual process is shown in Figure 1. What is worth mentioning is that the initial cytopathic effect (CPE) in primary neurons started to occur at 276 h post-infection (h p.i.) (Figure 1A, red arrow) by syncytium formation. After 454 h p.i. it was possible to distinguish two growing plaques, but the cell culture confluence was still 82% (97,66% before infection, Figure 1B). The progressive changes started to appear after 577 h p.i. when more plaques were formed (Figure 1A, red arrow), and cells’ confluence decreased to 78,5% (Figure 1B). Within a few hours, there was visible cell degradation along with extensive plaque formation. These events resulted in decreasing cell confluence to 23,9% after 628 h p.i. (Figure 1B). It was notable that some neurons survived the infection (Figure 1A, orange arrow). Their activity was sustained until the end of the assay three days later.

**Figure 2:**
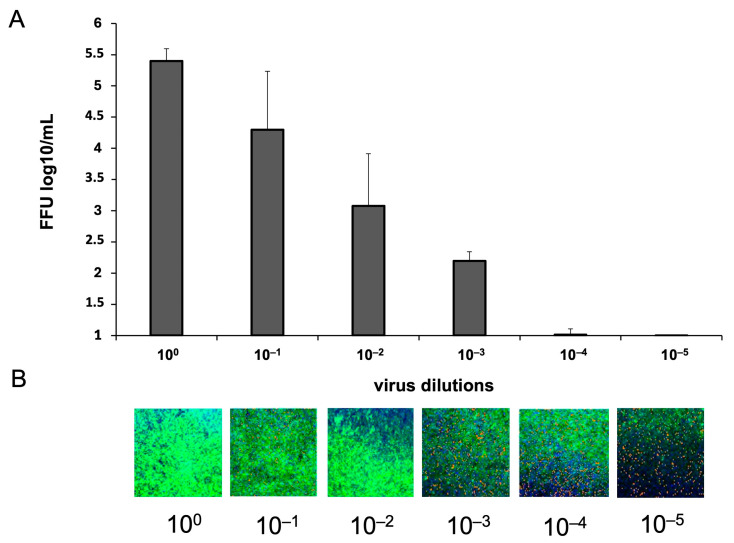
Virus titer is defined as log10 FFU/mL in neurons infected with 10-fold dilutions of SDAV stock solution (**A**). Representative images of individual wells of a 96-well plate seeded with primary neuron cells infected with 10-fold dilutions of SDAV. SDAV nucleocapsid protein (green), cell nuclei (blue) (**B**). Array Scan XTI (ThermoFisher™, Waltham, MA, USA), ×5 magnification.

The Determination of fluorescent focus units (FFU) in cultured primary neurons was performed using Array Scan XTI (ThermoFisher™, Waltham, MA, USA). Figure 2B shows images from individual wells of a 96-well plate where primary neurons infected with 10-fold dilutions of SDAV (10^0^–10^−5^) were cultured. SDAV titer was determined as log10 FFU/mL = 2.193 ± 0.15 (Figure 2A).

### 2.2. Evaluation of SDAV Replication in Primary Murine Neurons

**Figure 3:**
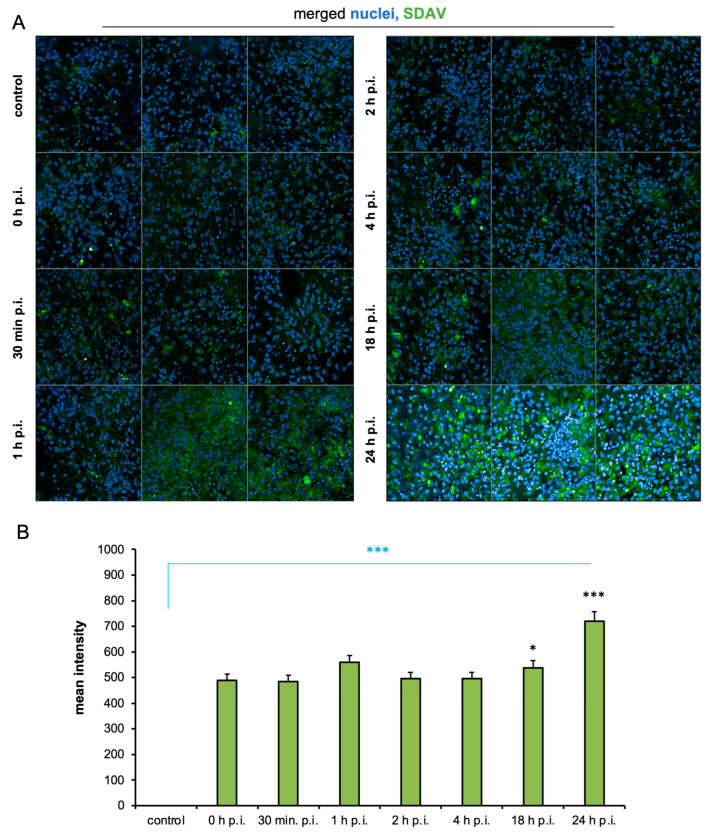
Representative images for each time point after SDAV infection (0–24 h). Cell nuclei (blue), SDAV antigen (green). Magnification x10 (**A**). Mean fluorescence intensity corresponding to virus protein as a function of time after infection of primary neuron cells (**B**). Negative control—uninfected cells. Data from three independent experiments are shown as mean ± SD. One-way ANOVA, * *p* < 0.05, *** *p* ≤ 0.001. Blue asterisk—comparison to uninfected control; black asterisk—comparison to 0 h p.i.

The visualization of SDAV nucleocapsid proteins and cellular structures in primary neuron cells made it possible to assess viral replication using high-content image analysis. An analysis of the images showed the presence of signals corresponding to the nucleocapsid protein inside infected cells (Figure 3A). No signal specific to the nucleocapsid protein was observed in control cultures. In images showing 0 h p.i., 0.5 h p.i., 2 h p.i., and 4 h p.i. cells, a similar intensity of green signal was observed. Images taken of 1 h p.i. and 18 h p.i. neurons showed higher levels of green fluorescence compared to the aforementioned post-infection times. The highest increase was observed in images showing 24 h p.i. cells (Figure 3A). A quantitative analysis was performed on an average of 400 cells per field of view. When comparing the mean fluorescence intensity corresponding to the SDAV nucleocapsid protein at different times after infection (0–24 h p.i.) to that of the negative control, a highly statistically significant increase in fluorescent intensity was detected at all tested times (Figure 3B, blue asterisk). The smallest increase was observed at 0 and 0.5 h p.i., while the largest increase was seen at 24 h p.i. Comparison to 0 h p.i. showed a statistically significant increase only at 18, 24 h p.i. (Figure 3B, black asterisks)

**Figure 4:**
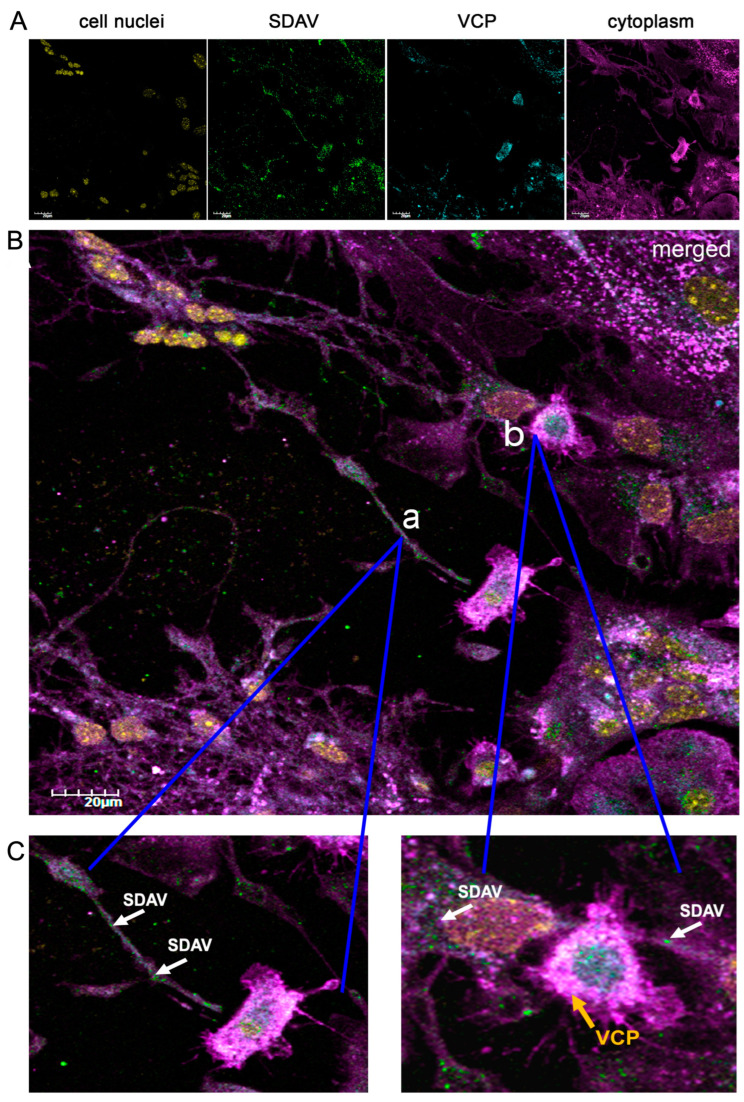
Representative confocal images of primary neurons morphology after infection with SDAV for 24 h (**A**). Merged image of SDAV antigen (green), VCP (blue), cell nuclei (yellow), cell membrane (magenta) (**B**). Closeup image of SDAV antigen moving inside cell protrusion (**C**(**a**)). Closeup of viral antigen in close affinity of cell nuclei and possible colocalization with VCP (whitish fluorescence) (**C**(**b**)). Magnification ×60, scale bar 20 μm. Olympus FV10i.

Analyzing the morphology of SDAV-infected neurons, virus antigens can be seen in the area of the cell’s membrane and moving inside neurites after 24 h p.i. (Figure 4C(a)), white arrows). Furthermore, it is visible that the signal corresponding to the VCP antigen is in close affinity with the virus antigen (Figure 4C(b), yellow arrow, and white arrows).

### 2.3. Levels of VCP in SDAV-Infected Neurons

**Figure 5:**
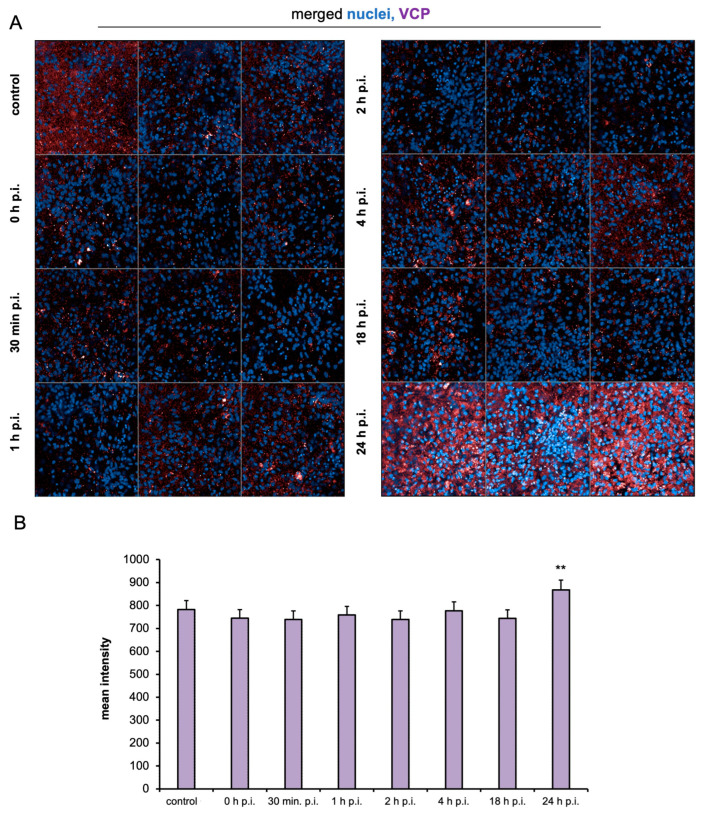
Representative images for each time point after SDAV infection (0–24 h). Cell nuclei (blue), VCP antigen (red). Magnification ×10 (**A**). Mean fluorescence intensity corresponding to VCP as a function of time after SDAV infection of primary neuron cells (**B**). Negative control—uninfected cells. Data from three independent experiments are shown as mean ± SD. One-way ANOVA, ** *p* ≤ 0.01.

Similarly, an HCS analysis of the mean fluorescence intensity signal of VCP antigen in the control and cells infected from 0 to 24 h showed interesting results. In the time from 0 to 18 h p.i., there was no significant increase in antigen levels compared to the uninfected control (Figure 5A,B). A rise in the mean intensity of the red fluorescence signal (VCP antigen) was observed in images taken 24 h p.i. A highly significant increase was detected in the quantitative analysis compared to the positive, uninfected control (Figure 5A). This was also observed in qualitative observation as a bright fluorescence signal of VCP antigen in the perinuclear area (Figure 4C, yellow arrows).

### 2.4. Evaluation of the Contribution of VCP to the SDAV Replication Cycle in Cultured Primary Neurons

**Figure 6:**
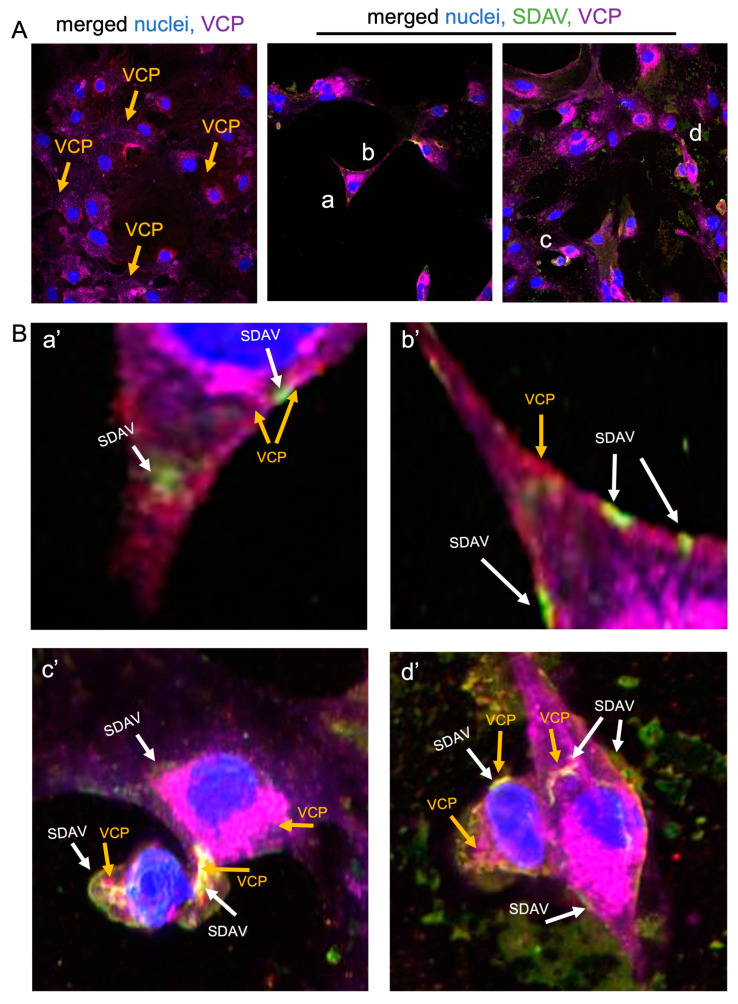
Representative confocal images of primary neurons morphology after infection with SDAV for 24 h treated with 5 μM/mL EerI and control nontreated. Merged image of SDAV antigen (green), VCP (orange), cell nuclei (blue), and cell membrane (magenta) (**A**,**B**; **a**–**d** are regions zoomed in and shown as **a’**–**d’**). Closeup image of SDAV antigen in the perinuclear area possibly correlating with VCP (whitish fluorescence) (**B**(**a’**,**c’**)). Closeup of viral antigen trapped in the submembrane region along with VCP (**B**(**b’**,**d’**)). Magnification ×60, scale bar 20 μm. Operetta CLS (Revvity™, Waltham, MA, USA).

**Figure 7:**
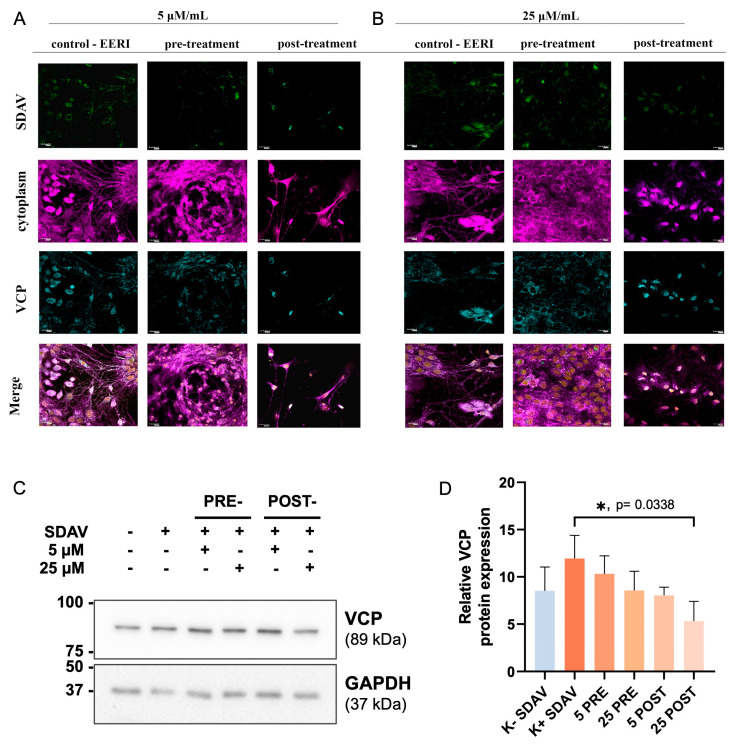
Representative images of neurons 24 h p.i. with SDAV. Cells were treated with 5 μM/mL and 25 μM/mL EerI in pre-incubation and post-incubation methods. Positive control—infected cells, untreated with EerI. Cytoplasm (magenta), VCP antigen (blue), SDAV nucleocapsid protein (green). FV10i (Olympus), magnification ×40, scale bar 20μm (**A**,**B**). Representative Western blot image for VCP followed by densitometric semi-quantification of relative VCP expression in infected neurons (SDAV+), pre- or post-incubated with VCP EerI inhibitor (5 µM/mL or 25 µM/mL separately), compared to non-infected control (SDAV−). Acquired in Image Lab™ Software v6.0.1 (Bio-Rad, Hercules, CA, USA) (**C**). Data from three independent experiments are presented as mean ± SD (**D**). Non-parametric, Friedman test, followed by post hoc group comparison test, * *p* < 0.05.

**Figure 8:**
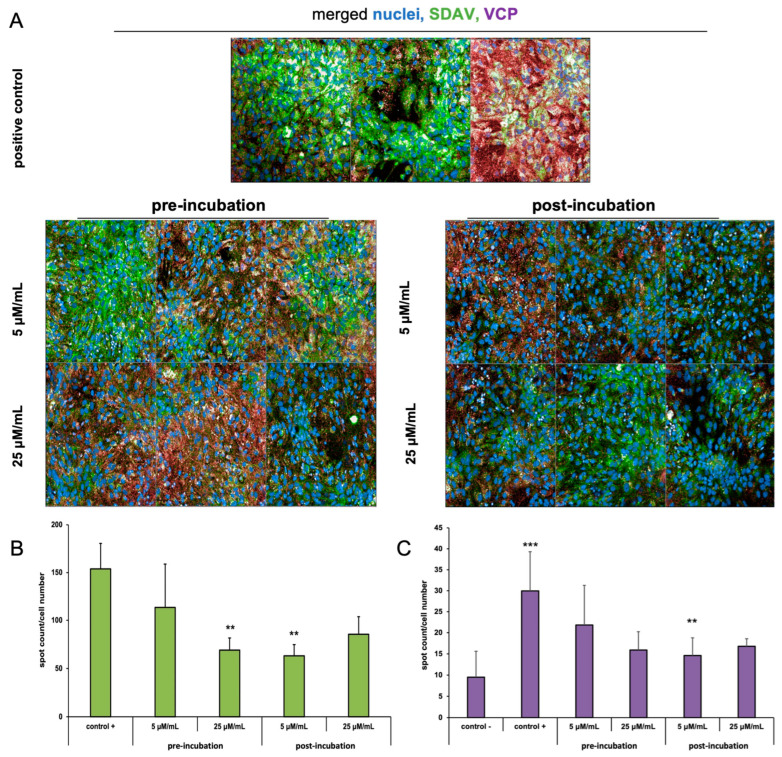
High-content imaging screening spot detection analysis of SDAV nucleocapsid and VCP antigen 24 h p.i in neurons. Representative images for infected untreated cells (positive control) and cells treated with EerI inhibitor at concentrations of 5 μM/mL and 25 μM/mL in the pre-incubation and post-incubation system. Cell nuclei (blue), viral antigen (green), VCP (red signal). Operetta^®^ CLS™ (Revivity™, Waltham, MA, USA), ×40 magnification (**A**). Quantitative analysis corresponding to the nucleocapsid protein of SDAV (**B**) and VCP (**C**) depending on the incubation method and inhibitor concentration using Harmony™ 4.9 software (Revvity™, Waltham, MA, USA) spot detecting protocol. Negative control—non-infected cells, positive control—infected, untreated cells. Data from three independent experiments are presented as mean ± SD. Two-way ANOVA, ** *p* ≤ 0.01 (to positive control) or *** *p* ≤ 0.001 (to negative control).

The potential involvement of VCP in the SDAV replication cycle was assessed by visualizing infected cultures untreated (Figure 4B) or treated with EerI (Figure 6 and Figure 7A,B) and by quantitative analysis of the spot count/cell of the VCP and N protein antigens in EerI-treated cultures compared to infected controls not treated with the inhibitor (Figure 8B,C).

The SDAV antigen is located in the outer cell membrane region after incubation with 5 μM/mL EerI (Figure 6B(b’,d’); white arrows). Nevertheless, in non-treated cells, the virus antigen is detected moving in cell protrusions (Figure 4C(a), white arrows). Interestingly, there is a noticeable, possible colocalization of VCP with SDAV antigens seen as bright magenta fluorescence (Figure 6B(c’,d’), yellow and white arrows).

The analysis of morphological alterations in cells revealed no abnormalities after administering 5 μM/mL and 25 μM/mL EerI for 24 h p.i. However, notable alterations in the intensity of the viral antigen and VCP fluorescence signals were observed following the treatment with both inhibitor concentrations, particularly in the post-incubation method. The fewest changes were observed in cultures that had been pre-incubated with 25 μM/mL EerI. In these cultures, the intensity of the fluorescence of the viral antigen and VCP was found to be similar to that observed in the control cells that had not been treated (Figure 7A,B). Western blot analysis demonstrated a statistically significant (*p* < 0.05) increase in VCP expression 24 h p.i. (12 ± 1.1) in comparison to the uninfected control cells (9 ± 0.98) (Figure 7C,D). Following incubation with EerI, a statistically significant (*p* < 0.05) decrease in VCP expression was observed in both incubation methods when compared to the positive infected control. Pre-incubation with 5 µM/mL (10 ± 1.1 vs. 12 ± 1.1), 25 µM (9 ± 0.95 vs. 12 ± 1.1), and post-incubation with 5 µM/mL (8.7 ± 0.4 vs. 12 ± 1.1), 25 µM (5 ± 1.0 vs. 12 ± 1.1) indicated a statistically significant (*p* < 0.05) drop in VCP expression. A decrease in VCP expression in comparison to the uninfected control was only observed for the post-incubation method at the 25 µM/mL concentration level (9 ± 0.98 vs. 5 ± 0.90).

The visualization of infected cultures treated with EerI along with quantitative analysis was performed using Operetta^®^ CLS™ and Harmony™ (Revvity™, Waltham, MA, USA) (Figure 8A–C). A quantitative analysis was performed for approximately 400 cells present in the field of view. The spot count of AlexaFluor™ 488 (N protein) and AlexaFluor™ 647 (VCP) signals in EerI-treated cultures was compared to the infected, non-EerI-treated neurons (positive control) after 24 h p.i. Changes in spot count number per cell corresponding to the nucleocapsid protein were detected for both incubation methods (5 µM/mL or 25 µM/mL) compared to positive control (153.87 ± 26.7) (Figure 8B). A highly statistically significant (** *p* ≤ 0.01) decrease in the spot count of N protein occurred in pre-incubated 25 μM/mL (69.07 ± 12.5) and post-incubated 5 μM/mL (63.17 ± 11.5) EerI-treated neurons (Figure 8B).

Interestingly the spot number of VCP increased extremely statistically significantly (*** *p* ≤ 0.001) after SDAV infection (29.96 ± 9.35) compared to the uninfected, untreated control (9.52 ± 6.1) (Figure 8C). After treatment with EerI, a highly significant statistical decrease (** *p* ≤ 0.01) in the spot count of VCP was observed only in the post-incubation method with 5 μM/mL EerI (14.61 ± 4.19). The decreasing trend of the VCP number was observed after SDAV infection compared to the positive control. The VCP number increased in all conditions when compared to the negative control (Figure 8C).

**Figure 9:**
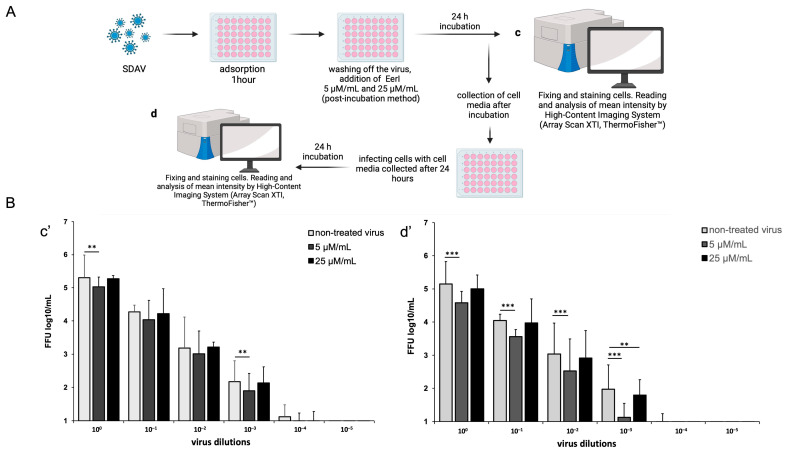
High-content analysis for SDAV nucleocapsid antigen after post-incubation with EerI at concentrations of 5 μM/mL and 25 μM/mL (HCS™ studio software v2.0 spot detector protocols, ThermoFisher^®^). Schematic representation of the procedure (**A**; **c**,**d** represent analysis steps with respective results shown in **c’**,**d’**). Mean fluorescent signal intensity of spots detected for virus antigen in the function of 10-fold dilutions of SDAV stock solution (**B**(**c’**)). Mean fluorescent signal intensity of spots detected for virus antigen in the function of 10-fold dilutions of previously infected cell media to test the hindering effect of the EerI inhibitor on the release of SDAV progeny virions (**B**(**d’**)). Data from three independent experiments are presented as mean ± SD. Two-way ANOVA, ** *p* ≤ 0.01, *** *p* ≤ 0.001.

The changes in SDAV titer following treatment with EerI were evaluated using high-content analysis (HCA), with the results expressed as log10 FFU/mL. To evaluate the efficacy of the employed treatment, the SDAV was initially titrated with the EerI inhibitor at concentrations of 5 μM/mL and 25 μM/mL in a post-incubation system (chosen based on previous promising results) for 24 h p.i. (Figure 9A(c)). Subsequently, prior to analysis, the cell media was collected and utilized for a second titer analysis to ascertain whether EerI influenced SDAV egress from neurons (Figure 9A(d)).

A highly statistically significant (** *p* ≤ 0.01) decrease in log10 FFU/mL occurred in a concentration of 5 μM/mL EerI by 0.278 logarithms in dilution 10^0^ and 0.270 logarithms in dilution 10^−3^ compared to the non-treated virus (dilution 10^0^: 5.39 ± 0.81 non-treated vs. 5.03 ± 0.29 5 μM/mL treated and 10^−3^: 2.19 ± 0.51 non-treated vs. 1.90 ± 0.51 5 μM/mL treated) (Figure 9B(c’)). Interestingly, an extremely significant decrease (*** *p* ≤ 0.001) in log10 FFU/mL appeared in the second round of viral tittering with collected cell media (Figure 9B(d’)). Again, the best results were obtained after incubation with 5 μM/mL EerI, where a 0.840 logarithms drop in dilution 10^−3^ was observed (1.96 ± 0.53 non-treated vs. 1.12 ± 0.41 5 μM/mL treated).

**Figure 10:**
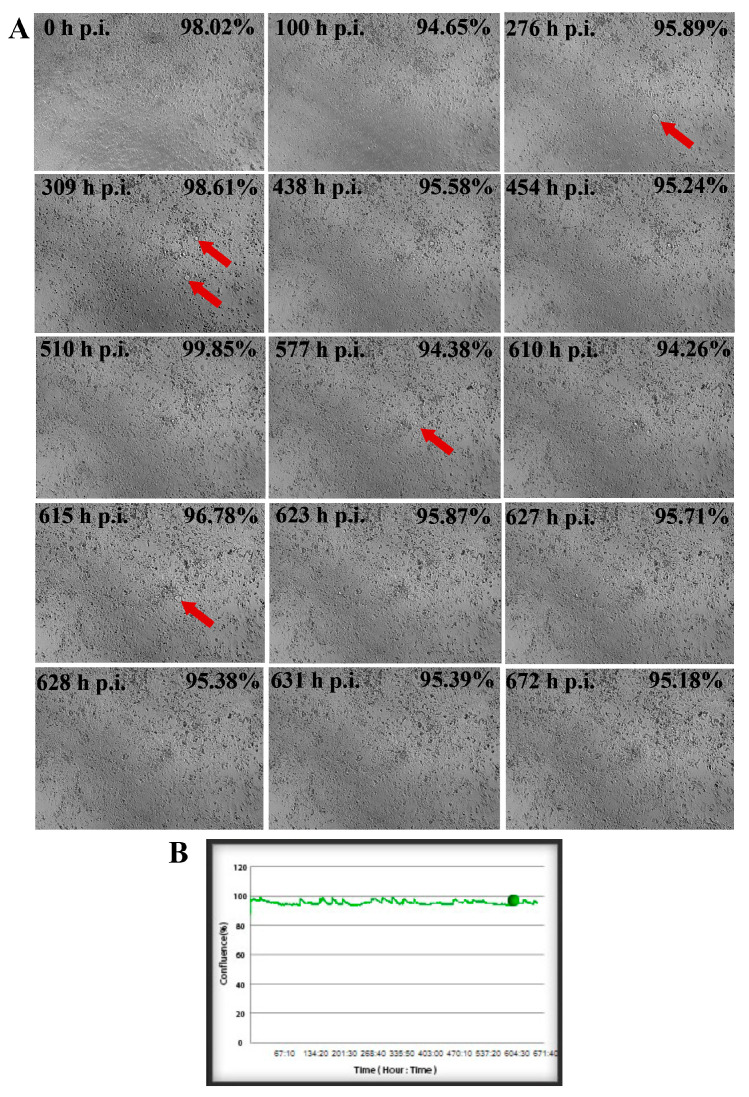
Real-time cell growth analysis of SDAV-infected primary murine neurons post-incubated with EerI at a concentration of 5 μM/mL performed by live image move analyzer JuLi™Br. Cultures were observed for 672 h p.i. Red arrows indicate the process of creating the CPE (**A**). The generated graph shows the percentage of cells’ confluence level [%] during the whole analysis [hours]. Images were taken every 10 min and analyzed for cell confluence (**B**). Objective magnification ×40.

To assess the morphology of cells and the potential manifestation of CPE following SDAV infection, a post-incubation method utilizing EerI at a concentration of 5 μM/mL was performed. This employed additional real-time growth analysis over a 672 h interval (Figure 10). During the course of the infection, the formation of small plaques was observed, though they did not evolve into larger plaques (Figure 10A, red arrows). The level of cell confluence remained above 94% throughout the entire recording span, indicating that EerI had a limiting effect on the egress of SDAV progeny virions (Figure 10B).

## 3. Discussion

The Sialodacryoadenitis virus, a highly infectious pathogen that causes infections in rats, poses a potential threat to humans and other animals due to possible interspecies transfer. Cases of mutation of the genome of various coronaviruses leading to the acquisition of the ability to infect new species have been documented [4]. It is now known that VCP can be a pro- or antiviral factor. Its effect has so far been verified for picornaviruses [32,46,47], flaviviruses [48,49,50], and coronaviruses (IBV, HCoV 229E, HCoV OC43) [41,43]. For this reason, the present study decided to carry out research to gain a better understanding of SDAV. We decided to target valosin-containing protein, knowing its crucial role in cell metabolism and viral replication. A successful in vitro multiplication of the Sialodacryoadenitis virus has so far been found in a few established lines (L2p, LBC) and a primary culture of rat kidney cells [5,51,52,53]. An attempt to multiply SDAV in mice brain cells’ primary culture was unsuccessful [5], despite studies showing brain lesions in neonatal CD-1, CWF mice, and W1 Wistar rats infected with SDAV [10].

For the first time, we demonstrated SDAV replication in the primary neuron culture of BALB/c mice without previous adaptation. Focal and diffuse cytopathic effects were shown in primary neurons (Figure 1A, red arrows). The PFU = 10^6^ and log10 FFU/mL = 2.193 ± 0.15 obtained in the study are comparable to those obtained by Gaertner et al. (1992) [54] (Figure 2). The onset of plaque formation was observed at 276 h p.i. (Figure 1A, red arrows), while in the case of the infected LBC established line, defined plaques were visible after 48 h p.i. [55]. This may be an indication of the movement of SDAV virions between neurons without cell destruction. An analysis of SDAV replication in primary neuron cells derived from BALB/c mice was carried out based on the Harmony (Revvity™, Waltham, MA, USA) algorithm’s calculation of the average fluorescence intensity corresponding to the SDAV nucleocapsid protein (Figure 3). Compared to cells analyzed at 0 h p.i. (after cell entry), there was an extremely statistically significant (*** *p* ≤ 0.001) increase in the mean fluorescence intensity at 24 h p.i., confirming the effective penetration of SDAV into mouse neurons and the completion of a full SDAV replication cycle. To explore the role of VCP in SDAV replication, we first examined VCP levels in uninfected and SDAV-infected primary neurons. We reported a highly statistically significant increase (** *p* ≤ 0.01) in mean fluorescence intensity corresponding to VCP 24 h p.i., which may suggest increased production of this protein in neurons resulting from SDAV infection (Figure 5). Similar results were obtained from Western blot analysis, where there was a statistically significant (* *p* ≤ 0.05) increase in relative VCP expression in infected cells (12 ± 1.1) in comparison to the uninfected control cells (9 ± 0.98) (Figure 7C,D).

The effect on VCP expression was studied before in the context of SARS-CoV and CoV-229E infection. It was shown that 24 h p.i. VCP expression in human monocytes for both coronaviruses decreased [56], contrary to our results. Coronaviruses require a suitable environment for replication to take place, which is created, among other things, by DMVs, which are thought to originate from EDEMosomes—vesicles that arise from the endoplasmic reticulum membrane and contain regulators of the ERAD pathway. It has been shown that during betacoronavirus infection, there is increased formation of EDEMosomes. This may be related to the prevention of the accumulation of viral proteins in the ER, as regulators contained in EDEMosomes may increase ERAD activity and thus affect the release of viral structural proteins [57,58,59,60]. It is possible that the increase in the mean fluorescence corresponding to VCP and protein relative expression, one of the components of ERAD, is related to the activation of ERAD by regulators contained in EDEMosomes (Figure 5 and Figure 7C,D).

To further explore the role of VCP in SDAV replication, we used Eeyarestatin I (EerI), a VCP inhibitor, and checked its effect on SDAV-infected primary neurons. EerI is a substance that shows affinity for the endoplasmic reticulum due to its aromatic domain. EerI has been shown to interact with VCP located within the endoplasmic reticulum via a nitrofuran-containing group (NFC). The VCP present in this area is part of ERAD, a pathway associated with protein degradation [50,61]. In high-content screening analysis (Operetta CLS, Revvity™, Waltham, MA, USA) of the spot count/cell of the VCP antibody signal, an extremely statistically significant (*** *p* ≤ 0.001) increase in the infected, non-treated control was observed 24 h p.i. compared to uninfected neurons (29.96 ± 9.35 vs. 9.52 ± 6.1, respectively) (Figure 8C). The statistically significant decrease compared to the positive control occurred only in 5 μM/mL EerI post-treatment (29.96 ± 9.35 vs. 14.61 ± 4.19, respectively) (Figure 8C). No statistically significant changes were observed under other incubation conditions. This may be due to the action of EerI, its affinity for the endoplasmic reticulum, and its inability to interact with VCPs located in other organelles or in the cytoplasm [62]. In addition, an overall higher spot count/cell (Figure 8C) and a higher relative expression (Figure 7C,D) of VCP in infected neurons, both untreated and treated with EerI, suggest its important involvement in SDAV virion assembly. In the context of the SDAV N protein signal after EerI treatment, a highly statistically significant (** *p* ≤ 0.01) decrease in the spot count/cell was observed for 25 μM/mL pre-incubation (69.07 ± 12.5) and 5 μM/mL post-incubation (63.17 ± 11.5) compared to the positive control (153.87 ± 26.7) (Figure 8B). This indicates the likely involvement of VCP at a later stage of replication, during the release of viral proteins from the endoplasmic reticulum. An interesting observation that confirms the VCP role at later stages of viral replication was the accumulation of the viral antigen signal in the submembrane areas of neurons after post-incubating with 5 μM/mL (Figure 6B(b’,d’), white arrows). No presence was detected in cell protrusions like it was seen in the untreated infected control (Figure 4C, white arrows). Here, we can speculate that a similar phenomenon of “viral protein homeostasis” described by Tabata et al. (2022) [62] played a role by controlling the amount of each SDAV protein in virus-infected cells by the ERAD system modulated by the EerI inhibitor. Another study demonstrated that the administration of either Xanthohumol or Eeyarestatin I resulted in a reduction in Zika virus (ZIKV) and Usutu virus (USUV) titers in infected cells. This finding aligns with the crucial role of valosin-containing protein (VCP) during the intracellular stages of the viral replication cycle. Of particular interest, the research also revealed a previously unappreciated direct antiviral activity of Eeyarestatin I against virus infectivity (virucidal activity). However, this activity was exclusive to Eeyarestatin I, and not observed with Xanthohumol [63]. Concluding the results, we noticed a better effect in reducing virus yield by using the post-incubation method (Figure 6, Figure 7, Figure 8 and Figure 9). To evaluate the efficiency of this treatment, the SDAV was initially tittered with the EerI inhibitor at concentrations of 5 μM/mL and 25 μM/mL and, again, using a collected cell medium (after 24 h course of infection) to ascertain whether VCP inhibition by EerI influenced SDAV egress from neurons (Figure 9A,B). The analysis confirmed our hypothesis, indicating that the second titer showed an extremely statistically significantly (*** *p* ≤ 0.001) reduction in the virus titer.

The usage of 5 μM/mL decreased log10 FFU/mL by 0.840 logarithms in dilution 10^−3^ (1.96 ± 0.53 non-treated vs. 1.12 ± 0.41 5 μM/mL treated) (Figure 9B(d’)). This outcome prompted the decision to conduct the final analysis using only a concentration of 5 μM/mL in the post-treatment system. The real-time observation of neuronal growth following treatment and infection with SDAV for 672 h demonstrated a minimal cytopathic effect and a sustained cell confluence of no less than 94% (Figure 10). A comparison of the results of the same analysis conducted without prior incubation with EerI confirmed the effect of VCP on the assembly and release of SDAV progeny virions. In cells that had not been treated and that had been infected for 672 h, the level of confluence dropped to an incomplete 6%, and a significant cytopathic effect was observed, manifesting as plaques and cell vacuolization (Figure 10).

## 4. Materials and Methods

### 4.1. Primary Neuronal Cell Culture

Balb/c (H2^d^) mice were used to establish the primary culture of murine neurons using the method by Cymerys et al. (2010; 2016) [64,65]. Pregnant female mice (16–19 days post-mating) were sacrificed, and fetuses were removed and decapitated for brain collection. Then, isolated cerebral hemispheres from fetal brains were washed three times in cold HBSS solution (10x Hanks Buffer; Life Technologies Waltham, MA, USA) and treated with 2.5% EDTA-free trypsin solution at 37 °C in 5% CO_2_ for 15 min. Again, after incubation, cells were washed three times in a warm HBSS solution and mechanically homogenized using a pipette. After suspending and counting, cells were plated onto poly-L-lysine-coated wells or poly-D-lysine with laminin-coated coverslips. Primary murine neurons were cultured in B-27 Neuron Plating Medium, consisting of the neurobasal medium, B-27 supplement, 200 mmol/L of glutamine, 10 mmol/L of glutamate, 1% penicillin/streptomycin antibiotics with 5% supplement of fetal bovine, and 5% horse serum (Gibco Life Technologies, Waltham, MA, USA). To avoid propagation of non-neural cells, cultures were maintained in growth medium supplemented with 10 μM cytosine β-D-arabinofuranoside (after 3 days for 24 h) (Sigma-Aldrich, Darmstadt, Germany). Subsequently, the medium was removed and replaced with Neuron Feeding Medium (B-27 Neuron Plating Medium without glutamate; Life Technologies Waltham, MA, USA). In such conditions, neurons were maintained for the next 8 days before analysis, infection, and incubation with inhibitor at 37 °C with 5% CO_2_.

### 4.2. SDAV Infection and Calculation of Viral Antigen Signal Using High-Content Analysis

**Figure 11:**
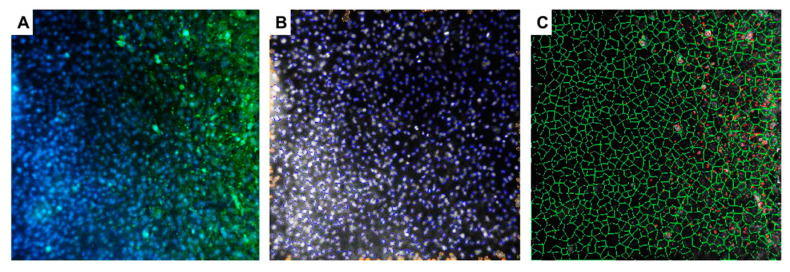
Representative images of SDAV-infected primary neurons presenting how the algorithm for detecting the spot-protein area of the SDAV nucleocapsid works. (**A**) Overlay of green signal (AlexaFluor™ 488, SDAV N protein) and blue signal (Hoechst 33258, cell nuclei), (**B**) non-blue signals (cell nuclei) from which the algorithm determines the cell area, (**C**) green lines represent cell areas, and red dots represent the detected viral antigen: Array Scan XTI (ThermoFisher™, USA), magnification ×5.

The virus used in the study, Sialodacryoadenitis virus strain 681, was provided courtesy of Professor Susan Compton, Yale University, USA. The strain was isolated in 1976 at Yale [66,67]. A rat L2 lung epithelial cell line (CCL-149™, ATCC^®^, Manassas, VA, USA) was used to multiply and determine the viral titer (PFU/mL = 10^6^ using the method by Gaertner et al. (1993)) [54]. Viral titer in primary murine neurons was assessed by the fluorescent focus units (FFU). Cells were seeded in 96-well plates (at a density of 5 × 10^3^ cells/well) and infected with 10-fold dilutions of the SDAV (from 10^0^–10^−5^ in twelve replicates). After 1 h of incubation in 5% CO_2_, 37 °C, the virus suspension was aspirated, and a fresh growth medium was added. At 24 h post-infection (h p.i.), cells were fixed in 3.7% paraformaldehyde (PFA) in PBS (ThermoFisher™, USA) for 15 min at room temperature (RT). Next, the cells were permeabilized with 0.5% Tween (Sigma-Aldrich) in PBS (15 min, RT) and blocked with 1% bovine serum albumin (BSA, Sigma-Aldrich) in PBS (30 min, RT) to prevent nonspecific binding. Next, SDAV nucleocapsid proteins were visualized by incubation with mouse-SARS/SARS-CoV-2 monoclonal primary antibody specific for N protein (ThermoFisher™) (1:250 dilution, 1 h, 37 °C) and AlexaFluor™ 488 Goat anti-Mouse IgG secondary antibody (ThermoFisher™) (1:500 dilution, 1 h, RT). Cell nuclei were visualized for cell localization with Hoechst 33,258 (ThermoFisher™) (2 μg/1 mL, 3 min, RT). The fluorescent signals were detected via high-content analysis (Array Scan XTI, ThermoFisher™, Waltham, MA, USA) at ×10 magnification. The number of infected cells in each well was automatically obtained from 9 images per well (approx. 10,000 cells) using HCS studio software version 2.0 spot detector protocols. How the algorithm worked is shown in Figure 11. Results were presented as the number of spots detected (AlexaFluor 488™) corresponding to virus antigen in log10 FFU/mL [68,69].

### 4.3. Real-Time Imaging of SDAV Cytopathic Effect in Primary Neurons

To determine the cellular growth and morphology of primary neurons infected with SDAV and incubated with VCP inhibitor, the JuLI^TM^Br Live Cell-system for bright-field analysis (NanoEnTek, Seoul, Republic of Korea) was used [70]. When cultured neurons reached about 90% confluency, cells were infected with undiluted SDAV suspension (10^0^), as previously described. Images were captured for 672 h with 10 min intervals. The results were obtained and analyzed using JuLi^TM^Br PC v1.01. software. Uninfected cells were used as a negative control. All images were captured at ×40 magnification.

### 4.4. Cell Treatment with VCP-Interfering Inhibitor

Eeyarestatin I (EerI, Sigma-Aldrich^®^) was used to determine the potential use of VCP by SDAV in the replication cycle. It is a membrane-penetrating substance that preferentially localizes near the endoplasmic reticulum. EerI causes inhibition of the ERAD pathway through an irreversible interaction with VCP. Cell viability after EerI treatment was detected by XTT assay (data not shown) (The Cell Proliferation Kit II (XTT), Roche, Basel, Switzerland). EerI concentrations were chosen by XTT assay results and literature data [63,71,72]. The potential use of VCP by SDAV was investigated by treating primary neuron cultures with EerI before and after virus infection (pre-incubation and post-incubation, respectively). All analyses of EerI-treated cultures were performed in triplicates in a 96-well plate and Nunc™ systems. Positive controls were infected cells not treated with EerI. Noninfected cells were negative control. Pre-incubation consisted of adding EerI diluted in culture medium (5 μM/mL and 25 μM/mL, 1 h, 5% CO_2_, 37 °C) to uninfected primary neuron cultures, aspiring the EerI media, adding the SDAV to the cell culture (1 h, 5% CO_2_, 37 °C), and replacing inoculum with fresh medium. Post-incubation, on the other hand, consisted of infecting the primary neuron culture with the SDAV (1 h, 5% CO_2_, 37 °C), washing of the SDAV, and adding EerI in growth medium (5 μM/mL and 25 μM/mL, 24 h, 5% CO_2_, 37 °C). After 24 h, cultures were fixed and prepared for visualization as described above.

### 4.5. SDAV Titration After EerI Treatment Using High-Content Analysis

To determine the outcome of EerI treatment on viral titer, we repeated viral titration using focus forming assay. Primary neuron cultures were post-incubated with 5 μM/mL or 25 μM/mL EerI and fixed after 24 h of incubation (5% CO_2_, 37 °C). As described above, fluorescence staining of N protein and cell nuclei and high-content screening were carried out. To evaluate the efficacy of the employed treatment, the SDAV was initially titrated with the EerI inhibitor at concentrations of 5 μM/mL and 25 μM/mL in a post-incubation system for 24 h p.i. Subsequently, prior to analysis, the cell media was collected and utilized for a second titer analysis to ascertain whether EerI influenced SDAV egress from neurons. A schematic representation is shown in Figure 9A, which was made with Biorender [73].

### 4.6. Immunofluorescence Staining for Morphology Analysis of SDAV Infected Neurons

The immunofluorescence method was used to visualize cell structures and viral antigens. After pre-incubation and post-incubation with 5 μM/mL or 25 μM/mL EerI, primary neuronal cell cultures were washed twice in PBS (Sigma-Aldrich, Darmstadt, Germany), then fixed in 4% PFA (Thermo Fisher, Waltham, MA, USA) for 10 min at 24 h p.i. After fixation, the cells were washed twice with PBS solution and incubated with 1% Tween/PBS solution for 5 min at room temperature. Cells were then washed twice with PBS solution. After blocking with 1% BSA/PBS for 15 min, the cells were incubated with a 1:250 dilution ratio of primary antibody specific for SARS-CoV-2 Nucleoprotein (N) (Mouse mAb) (Thermo Fisher Scientific, Waltham, MA, USA) overnight at 4 °C. The unbound antibody was removed by washing with PBS three times. Then, the Goat anti-Mouse IgG (H + L) Highly Cross-Adsorbed Secondary Antibody, Alexa Fluor 488 (Thermo Fisher Scientific, USA), was used at a 1:1000 dilution ratio for 1h at room temperature. VCP was visualized by incubation with rabbit anti-VCP primary monoclonal antibody (ThermoFisher™) (1:500 dilution, overnight, 4 °C) and Texas Red™-X secondary goat anti-rabbit IgG antibody (ThermoFisher™) (dilution 1:500, 1 h, RT). To stain the cell membranes, cells were incubated with wheat germ agglutinin (WGA) conjugate, AlexaFluor™ 647 (ThermoFisher™) (10 μg/mL, 1 h, RT). Additionally, cell nuclei were stained with Hoechst 33,258 (Thermo Fisher, Waltham, MA, USA) for 2 min, RT. Afterward, cover slips were mounted on microscope slides using ProLong Gold Antifade Mounting Medium (Thermo Fisher, Waltham, MA, USA). Images were acquired in a confocal microscope (Fluoview FV10i, Olympus, Japan), saved in 24-bit .tiff format, and analyzed using FV10i v4.1 software (Olympus), ImageJ2 (NIH Image, v1.53q, Bethesda, MD, USA), and Adobe Photoshop CS6 software (Adobe Systems Incorporated, v23.4.1, San Jose, CA, USA). During data processing to improve visualization of the structures, the fluorescence signals were changed to the following colours: Texas Red™—blue; AlexaFluor™ 647—purple; Hoechst™—yellow.

### 4.7. High-Content Imaging System for Quantitative SDAV Nucleoprotein and Valosin-Containing Protein (VCP) Detection

The high-content screening was performed on cells in the 96-well plate with two methods. First, screening was performed without treatment (uninfected and 0, 0.30, 1, 2, 4, 18, 24 h p.i.) to assess the level of proteins. Second, it was performed with pre-treatment and post-treatment (5 μM/mL or 25 μM/mL EerI, 24 h p.i.) in order to check the VCP involvement in SDAV replication. Fluorescent staining was carried out as described in the confocal imaging section.

The images were acquired using the Operetta CLS high-content imaging system (Revvity™, Waltham, MA, USA), equipped with an ×40 water objective lens. The exposure time for each channel was customized to optimize the signal-to-noise ratio, ensuring clear and accurate data capture. Illumination intensity was adjusted for optimal fluorescent signal capture, and at least 400 cells were analyzed per condition, providing a robust dataset for downstream analysis.

The analysis of cellular images was conducted using the Harmony 4.9 software (Revvity™, Waltham, MA, USA). This process involved a sequence of segmentation and feature extraction steps designed to quantify specific protein distributions within cells. The Hoechst channel was selected, and Harmony’s built-in algorithm was used to perform automatic nuclei segmentation. Parameters were adjusted to accurately detect the nuclei, ensuring that objects of irregular shapes were recognized correctly. WGA conjugated to AlexaFluor™ 647 (ThermoFisher™) was used to stain the cell membrane and cytoplasm. Alexa647 channel was employed to detect cytoplasmic regions surrounding the nuclei. Noise reduction techniques, such as Gaussian filtering, were applied to enhance signal-to-background contrast. Using Harmony’s segmentation algorithms, the cytoplasm was segmented by expanding outward from the previously segmented nuclei. Careful parameter tuning was conducted to prevent cytoplasmic overlap between adjacent cells. Objects touching the image boundary were excluded from further analysis to avoid artifacts from incomplete cell structures. Spot detection algorithms in Harmony were utilized to locate distinct fluorescent spots corresponding to VCP and SDAV proteins. Parameters such as spot size, intensity threshold, and minimum distance between spots were optimized for precise detection. The total number of spots per cell was calculated.

### 4.8. VCP Expression Analysis by Western Blot

Murine primary neuron cells were cultured and treated accordingly (5 μM/mL or 25 μM/mL EerI post- and pre-treated for 24 h p.i.). Cell lysates were prepared with cell lysis buffer (N-PER™ Neuronal Protein Extraction Reagent, Thermo Scientific™, USA) and protease inhibitor (Halt™ Protease Inhibitor Cocktail, Thermo Scientific™, USA). Protein content was measured using Micro BCA™ Protein Assay Kit (Thermo Scientific™, USA), and protein samples were prepared using an equal amount of protein, 4× Laemmli Sample Buffer (Bio-Rad, USA) and 2-mercaptoethanol. Protein samples were heated at 95 °C for 10 min and equally loaded and separated on 12% polyacrylamide gels by electrophoresis (Mini-PROTEAN Tetra Cell, Bio-Rad, USA) in the following conditions: 80 V for 15 min, followed by 120 V for 85 min and then transferred to polyvinylidene difluoride (PVDF) 0.45 µm membranes (Immobilon^®^ P Membrane, Merck Millipore, Burlington, MA, USA) using wet-blotting system (Bio-Rad, USA) and 330 mA for 1h in cooling conditions. Membranes were blocked after transfer for 30 min in 5% skim milk in TBS-Tween 20 (TBS-T) buffer at room temperature and incubated overnight with primary antibodies at 4 °C. The following day, membranes were washed 5 times for 5 min in TBS-T buffer and placed in secondary antibodies conjugated with horseradish peroxidase for 1 h at room temperature. After incubation, membranes were washed again using the same protocol, and proteins were visualized with an ECL kit (Clarity™, Bio-Rad, USA) using ChemiDoc Touch Imaging System (Bio-Rad, USA) using signal accumulation mode (SAM) with the same settings for each membrane (Bio-Rad, USA). Densitometry was performed using images acquired after the exact same time of exposure in Image Lab™ Software v6.0.1 (Bio-Rad, USA). Each target protein band intensity was compared to the intensity of the GAPDH protein band (loading control). Expression is presented as a relative protein expression (target protein/loading control). All antibodies used were diluted in blocking buffer, and the following dilutions were used: anti-VCP (1:1000, #MA3-004, Invitrogen, ThermoFisher, USA), anti-GAPDH (1:5000, #MA5-15738, Invitrogen, ThermoFisher, USA). Secondary HRP-conjugated antibodies were used, including anti-mouse (1:5000, #31450, Invitrogen, ThermoFisher, USA). Antibodies were freshly prepared and used once.

### 4.9. Statistical Analysis

The results were statistically evaluated by one-way or two-way analysis of variation (ANOVA) using Tukey’s multiple comparisons test or multiple unpaired t-test using threshold *p*-value with the Šídák–Bonerroni multiple comparisons correction method. For Western blot analysis, the non-parametric Friedman test, followed by a post hoc group comparison test, was used. All experiments were performed at least in triplicate. These analyses were performed using GraphPad Prism^TM^ version 9.4.0 (453) for macOS software (GraphPad Software Inc., San Diego, CA, USA). Statistical differences were interpreted as significant at *p* ≤ 0.05, highly significant at *p* ≤ 0.01, extremely significant at *p* ≤ 0.001, and not significant at *p* > 0.05.

## 5. Conclusions

In addition to its multiple roles in regulating cellular homeostasis, particularly in nervous system cells, the ATPase valosin-containing protein is an important host factor in viral infections. It remains unclear in which critical replication points VCP is used by SDAV. However, the results of our study have provided insight into the important role of the VCP in the assembly and release of progeny virion. We have shown for the first time that SDAV infection in neurons enhances VCP expression, which explains the use of ATPase in the viral replication cycle. In addition, using an EerI inhibitor that targets the role of VCP in the ERAD system results in an apparent reduction in viral titer. These changes are particularly evident in the second round of titers replicated from the cell medium after the first infection cycle. What is more, based on confocal image analysis, we can conclude that EerI influences the SDAV capability of viral protein assembly and egress from the cell by “trapping” the virions in the submembrane area.

## Data Availability

The data used to support the findings of this study are included in the article.

## References

[1] Decaro N., Lorusso A. (2020). Novel human coronavirus (SARS-CoV-2): A lesson from animal coronaviruses. Vet. Microbiol..

[2] Lau S.K., Woo P.C., Li K.S., Tsang A.K., Fan R.Y., Luk H.K., Cai J.P., Chan K.H., Zheng B.J., Wang M. (2015). Discovery of a novel coronavirus, China Rattus coronavirus HKU24, from Norway rats supports the murine origin of Betacoronavirus 1 and has implications for the ancestor of Betacoronavirus lineage A. J. Virol..

[3] Bartak M., Słońska A., Bańbura M.W., Cymerys J. (2021). SDAV, the Rat Coronavirus-How Much Do We Know about It in the Light of Potential Zoonoses. Viruses.

[4] Bonilauri P., Rugna G. (2021). Animal Coronaviruses and SARS-CoV-2 in Animals, What Do We Actually Know?. Life.

[5] Bhatt P.N., Percy D.H., Jonas A.M. (1972). Characterization of the virus of sialodacryoadenitis of rats: A member of the coronavirus group. J. Inf. Dis..

[6] Parker J.C., Cross S.S., Rowe W.P. (1970). Rat coronavirus (RCV): A prevalent, naturally occurring pneumotropic virus of rats. Arch. Ges. Vir..

[7] Utsumi K., Ishikawa T., Maeda T., Shimizu S., Tatsumi H., Fujiwara K. (1980). Infectious sialodacryoadenitis and rat breeding. Lab Anim..

[8] Fauquet C.M., Fargette D. (2005). International Committee on Taxonomy of Viruses and the 3142 unassigned species. Virol. J..

[9] Kojima A., Fujinami F., Doi K., Yasoshima A., Okaniwa A. (1980). Isolation and properties of sialodacryoadenitis virus of rats. Exp. Anim..

[10] Percy D.H., Lynch J.A., Descôteaux J.P. (1986). Central nervous system lesions in suckling mice and rats inoculated intranasally with sialodacryoadenitis virus. Vet. Pathol..

[11] Ahlstedt B.A., Ganji R., Raman M. (2022). The functional importance of VCP to maintaining cellular protein homeostasis. Biochem. Soc. Trans..

[12] Meyer H., Weihl C.C. (2014). The VCP/p97 system at a glance: Connecting cellular function to disease pathogenesis. J. Cell Sci..

[13] Erzberger J.P., Berger J.M. (2006). Evolutionary relationships and structural mechanisms of AAA+ proteins. Annu. Rev. Biophys. Biomol. Struct..

[14] Ye Y., Tang W.K., Zhang T., Xia D.A. (2017). Mighty “Protein Extractor” of the Cell: Structure and Function of the p97/CDC48 ATPase. Front. Mol. Biosci..

[15] Calabrese G., Molzahn C., Mayor T. (2022). Protein interaction networks in neurodegenerative diseases: From physiological function to aggregation. J. Biol. Chem..

[16] Mee Hayes E., Sirvio L., Ye Y. (2022). A Potential Mechanism or Targeting Aggregates With Proteasomes and Disaggregases in Liquid Droplets. Front. Aging Neurosci..

[17] Buchan J.R., Kolaitis R.M., Taylor J.P., Parker R. (2013). Eukaryotic Stress Granules Are Cleared by Autophagy and Cdc48/VCP Function. Cell.

[18] Papadopoulos C., Kravic B., Meyer H. (2020). Repair or Lysophagy: Dealing with Damaged Lysosomes. J. Mol. Biol..

[19] Bhattacharya A., Qi L. (2019). ER-associated degradation in health and disease—From substrate to organism. J. Cell Sci..

[20] Joazeiro C.A.P. (2019). Mechanisms and functions of ribosome-associated protein quality control. Nat. Rev. Mol. Cell Biol..

[21] Escobar-Henriques M., Anton V. (2020). Mitochondrial Surveillance by Cdc48/ p97: MAD vs. Membrane Fusion. Int. J. Mol. Sci..

[22] Kilgas S., Ramadan K. (2023). Inhibitors of the ATPase p97/VCP: From basic research to clinical applications. Cell Chem. Biol..

[23] Chu S., Xie X., Payan C., Stochaj U. (2023). Valosin containing protein (VCP): Initiator, modifier, and potential drug target for neurodegenerative diseases. Mol. Neurodegeneration..

[24] Szczęśniak P.P., Heidelberger J.B., Serve H., Beli P., Wagner S.A. (2022). VCP inhibition induces an unfolded protein response and apoptosis in human acute myeloid leukemia cells. PLoS ONE.

[25] Zhang X., Jiang L., Li Y., Feng Q., Sun X., Wang Y., Zhao M. (2023). Discovery of novel benzylquinazoline molecules as p97/VCP inhibitors. Front. Pharmacol..

[26] Wei R., Cao Y., Wu H., Liu X., Jiang M., Luo X., Deng Z., Wang Z., Ke M., Zhu Y. (2023). Inhibition of VCP modulates NF-κB signaling pathway to suppress multiple myeloma cell proliferation and osteoclast differentiation. Aging.

[27] Valle C.W., Min T., Bodas M., Mazur S., Begum S., Tang D., Vij N. (2011). Critical Role of VCP/p97 in the Pathogenesis and Progression of Non-Small Cell Lung Carcinoma. PLoS ONE.

[28] Han D.Y., Di X.J., Fu Y.L., Mu T.W. (2015). Combining valosin-containing protein (VCP) inhibition and suberanilohydroxamic acid (SAHA) treatment additively enhances the folding, trafficking, and function of epilepsy-associated γ-aminobutyric acid, type A (GABAA) receptors. J. Biol. Chem..

[29] Das P., Dudley J.P. (2021). How Viruses Use the VCP/p97 ATPase Molecular Machine. Viruses.

[30] Lin Y.T., Prendergast J., Grey F. (2017). The Host Ubiquitin-Dependent Segregase VCP/P97 Is Required for the Onset of Human Cytomegalovirus Replication. PLoS. Pathog..

[31] Carissimo G., Chan Y.H., Utt A., Chua T.K., Bakar F.A., Merits A., Ng L.F.P. (2019). VCP/P97 Is a Proviral Host Factor for Replication of Chikungunya Virus and Other Alphaviruses. Front. Microbiol..

[32] Arita M., Wakita T., Shimizu H. (2012). Valosin-Containing Protein (VCP/P97) Is Required for Poliovirus Replication and Is Involved in Cellular Protein Secretion Pathway in Poliovirus Infection. J. Virol..

[33] Pleasure I., Black M., Keen J. (1993). Valosin-containing protein, VCP, is a ubiquitous clathrin-binding protein. Nature.

[34] Roy L., Bergeron J.J., Lavoie C., Hendriks R., Gushue J., Fazel A., Pelletier A., Morré D.J., Subramaniam V.N., Hong W. (2000). Role of p97 and syntaxin 5 in the assembly of transitional endoplasmic reticulum. Mol. Biol. Cell..

[35] Ramanathan H.N., Ye Y. (2012). The p97 ATPase associates with EEA1 to regulate the size of early endosomes. Cell Res..

[36] Chu J.J., Ng M.L. (2004). Interaction of West Nile virus with alpha v beta 3 integrin mediates virus entry into cells. J. Biol. Chem..

[37] Chu J.J.H., Ng M.L. (2004). Infectious Entry of West Nile Virus Occurs through a Clathrin-Mediated Endocytic Pathway. J. Virol..

[38] Xu Z., Waeckerlin R., Urbanowski M.D., van Marle G., Hobman T.C. (2012). West Nile virus infection causes endocytosis of a specific subset of tight junction membrane proteins. PLoS ONE.

[39] Ramanathan H.N., Zhang S., Douam F., Mar K.B., Chang J., Yang P.L., Schoggins J.W., Ploss A., Lindenbach B.D. (2020). A Sensitive Yellow Fever Virus Entry Reporter Identifies Valosin-Containing Protein (VCP/p97) as an Essential Host Factor for Flavivirus Uncoating. mBio.

[40] Liu D.X., Liang J.Q., Fung T.S. (2021). Human Coronavirus-229E, -OC43, -NL63, and -HKU1 (Coronaviridae). Enc. Virol..

[41] Wong H.H., Kumar P., Tay F.P., Moreau D., Liu D.X., Bard F. (2015). Genome-Wide Screen Reveals Valosin-Containing Protein Requirement for Coronavirus Exit from Endosomes. J. Virol..

[42] Anton A., Mazeaud C., Freppel W., Gilbert C., Tremblay N., Sow A.A., Roy M., Rodrigue-Gervais I.G., Chatel-Chaix L. (2021). Valosin-containing protein ATPase activity regulates the morphogenesis of Zika virus replication organelles and virus-induced cell death. Cell. Microbiol..

[43] Cheng K.W., Li S., Wang F., Ruiz-Lopez N.M., Houerbi N., Chou T.F. (2021). Impacts of p97 on Proteome Changes in Human Cells during Coronaviral Replication. Cells.

[44] Bojkova D., Klann K., Koch B., Widera M., Krause D., Ciesek S., Cinatl J., Münch C. (2020). Proteomics of SARS-CoV-2-infected host cells reveals therapy targets. Nature.

[45] Brahms A., Mudhasani R., Pinkham C., Kota K., Nasar F., Zamani R., Bavari S., Kehn-Hall K. (2017). Sorafenib Impedes Rift Valley Fever Virus Egress by Inhibiting Valosin-Containing Protein Function in the Cellular Secretory Pathway. J. Virol..

[46] Wu K.X., Phuektes P., Kumar P., Goh G.Y., Moreau D., Chow V.T., Chu J.J.H. (2016). Human genome-wide RNAi screen reveals host factors required for enterovirus 71 replication. Nat. Commun..

[47] Wang T., Wang B., Huang H., Zhang C., Zhu Y., Pei B., Cheng C., Sun L., Wang J., Jin Q. (2017). Enterovirus 71 protease 2Apro and 3Cpro differentially inhibit the cellular endoplasmic reticulum-associated degradation (ERAD) pathway via distinct mechanisms, nd enterovirus 71 hijacks ERAD component p97 to promote its replication. PLoS Pathog..

[48] Yi Z., Fang C., Zou J., Xu J., Song W., Du X., Pan T., Lu H., Yuan Z. (2016). Affinity purification of the hepatitis C virus replicase identifies valosin-containing protein, a member of the ATPases associated with diverse cellular activities family, as an active virus replication modulator. J. Virol..

[49] Phongphaew W., Kobayashi S., Sasaki M., Carr M., Hall W.W., Orba Y., Sawa H. (2017). Valosin-containing protein (VCP/p97) plays a role in the replication of West Nile virus. Vir. Res..

[50] Yi Z., Yuan Z. (2017). Aggregation of a hepatitis C virus replicase module induced by ablation of p97/VCP. J. Gen. Virol..

[51] Percy D., Bond S., MacInnes J. (1989). Replication of sialodacryoadenitis virus in mouse L-2 cells. Arch. Virol..

[52] Hirano N., Takamaru H., Ono K., Murakami T., Fujiwara K. (1986). Replication of sialodacryoadenitis virus of rat in LBC cell culture. Brief report. Arch. Virol..

[53] Hirano N., Suzuki Y., Ono K., Murakami T., Fujiwara K. (1986). Growth of rat sialodacryoadenitis viruses in LBC cell culture. Jpn. J. Vet. Sci..

[54] Gaertner D.J., Winograd D.F., Compton S.R., Paturzo F.X., Smith A.L. (1993). Development and optimization of plaque assays for rat coronaviruses. J. Virol. Methods.

[55] Hirano N. (1990). Plaque assay and propagation in rat cell line LBC cells of rat coronavirus and 5 strains of sialodacryoadenitis virus. J. Vet. Med. Ser. B.

[56] Hu W., Yen Y.T., Singh S., Kao C.L., Wu-Hsieh B.A. (2012). SARS-CoV regulates immune function-related gene expression in human monocytic cells. Viral Immunol..

[57] Movaqar A., Yaghoubi A., Rezaee S.R., Jamehdar S.A., Soleimanpour S. (2021). Coronaviruses construct an interconnection way with ERAD and autophagy. Future Microbiol..

[58] Noack J., Bernasconi R., Molinari M. (2014). How viruses hijack the ERAD tuning machinery. J. Virol..

[59] Reggiori F., Monastyrska I., Verheije M.H., Calì T., Ulasli M., Bianchi S., Bernasconi R., de Haan C.A., Molinari M. (2010). Coronaviruses Hijack the LC3-I-positive EDEMosomes, ER-derived vesicles exporting short-lived ERAD regulators, for replication. Cell Host Microbe.

[60] Reggiori F., de Haan C.A., Molinari M. (2011). Unconventional use of LC3 by coronaviruses through the alleged subversion of the ERAD tuning pathway. Viruses.

[61] Wang Q., Shinkre B.A., Lee J.G., Weniger M.A., Liu Y., Chen W., Wiestner A., Trenkle W.C., Ye Y. (2010). The ERAD inhibitor Eeyarestatin I is a bifunctional compound with a membrane-binding domain and a p97/VCP inhibitory group. PLoS ONE.

[62] Tabata K., Arakawa M., Ishida K., Kobayashi M., Nara A., Sugimoto T., Okada T., Mori K., Morita E. (2021). Endoplasmic Reticulum-Associated Degradation Controls Virus Protein Homeostasis, Which Is Required for Flavivirus Propagation. J. Virol..

[63] Rodrigo I., Ballesta C., Nunes E.B., Pérez P., García-Arriaza J., Arias A. (2022). Eeyarestatin I, an inhibitor of the valosin-containing protein, exhibits potent virucidal activity against the flaviviruses. Antivir. Res..

[64] Cymerys J., Dzieciątkowski T., Słońska A., Bierła J., Tucholska A., Chmielewska A., Bańbura M. (2010). Equine herpesvirus type 1(EHV-1) replication in primary murine neurons culture. Pol. J. Vet. Sci..

[65] Cymerys J., Słońska A., Skwarska J., Bańbura M.W. (2016). Function of myosin during entry and egress of equid herpesvirus type 1 in primary murine neurons. Acta Virol..

[66] Bihun C.G., Percy D.H. (1994). Coronavirus infections in the laboratory rat: Degree of cross protection following immunization with a heterologous strain. Can. J. Vet. Res..

[67] Zeiss C.J., Asher J.L., Vander Wyk B., Allore H.G., Compton S.R. (2021). Modeling SARS-CoV-2 propagation using rat coronavirus-associated shedding and transmission. PLoS ONE.

[68] Rak A., Matyushenko V., Prokopenko P., Kostromitina A., Polyakov D., Sokolov A., Rudenko L., Isakova-Sivak I. (2024). A novel immunofluorescent test system for SARS-CoV-2 detection in infected cells. PLoS ONE.

[69] Amarilla A.A., Modhiran N., Setoh Y.X., Peng N.Y.G., Sng J.D.J., Liang B., McMillan C.L.D., Freney M.E., Cheung S.T.M., Chappell K.J. (2021). An Optimized High-Throughput Immuno-Plaque Assay for SARS-CoV-2. Front. Microbiol..

[70] (2015). NanoEnTek Inc., Korea. http://www.nanoentek.com.

[71] Cross B.C., McKibbin C., Callan A.C., Roboti P., Piacenti M., Rabu C., Wilson C.M., Whitehead R., Flitsch S.L., Pool M.R. (2009). Eeyarestatin I inhibits Sec61-mediated protein translocation at the endoplasmic reticulum. J. Cell Sci..

[72] Wang Q., Li L., Ye Y. (2008). Inhibition of p97-dependent protein degradation by Eeyarestatin I. J. Biol. Chem..

[73] Bartak M. (2024). Created in BioRender. http://BioRender.com/g65u531.

